# Exploring the therapeutic potential of the mitochondrial transfer-associated enzymatic machinery in brain degeneration

**DOI:** 10.3389/fphys.2023.1217815

**Published:** 2023-07-28

**Authors:** Noymar Luque-Campos, Ricardo Riquelme, Luis Molina, Gisela Canedo-Marroquín, Ana María Vega-Letter, Patricia Luz-Crawford, Felipe A. Bustamante-Barrientos

**Affiliations:** ^1^ Laboratorio de Inmunología Celular y Molecular, Facultad de Medicina, Universidad de los Andes, Santiago, Chile; ^2^ Centro de Investigación e Innovación Biomédica, Universidad de los Andes, Santiago, Chile; ^3^ IMPACT-Center of Interventional Medicine for Precision and Advanced Cellular Therapy, Santiago, Chile; ^4^ Escuela de Nutrición y Dietética, Facultad de Medicina, Universidad de los Andes, Santiago, Chile; ^5^ Facultad de Medicina y Ciencia, Universidad San Sebastián, Puerto Montt, Chile; ^6^ Faculty of Dentistry, Universidad de los Andes, Santiago, Chile; ^7^ Escuela de Ingeniería Bioquímica, Pontificia Universidad Católica de Valparaiso, Valparaiso, Chile

**Keywords:** degenerative brain disorders, mitochondrial dysfunction, fission and fusion, mitophagy, oxidative damage, mitochondrial transfer, cellular therapy, enzymes

## Abstract

Mitochondrial dysfunction is a central event in the pathogenesis of several degenerative brain disorders. It entails fission and fusion dynamics disruption, progressive decline in mitochondrial clearance, and uncontrolled oxidative stress. Many therapeutic strategies have been formulated to reverse these alterations, including replacing damaged mitochondria with healthy ones. Spontaneous mitochondrial transfer is a naturally occurring process with different biological functions. It comprises mitochondrial donation from one cell to another, carried out through different pathways, such as the formation and stabilization of tunneling nanotubules and Gap junctions and the release of extracellular vesicles with mitochondrial cargoes. Even though many aspects of regulating these mechanisms still need to be discovered, some key enzymatic regulators have been identified. This review summarizes the current knowledge on mitochondrial dysfunction in different neurodegenerative disorders. Besides, we analyzed the usage of mitochondrial transfer as an endogenous revitalization tool, emphasizing the enzyme regulators that govern this mechanism. Going deeper into this matter would be helpful to take advantage of the therapeutic potential of mitochondrial transfer.

## 1 Introduction

Multiple mechanisms have been described in the onset and progression of degenerative brain disorders, such as defective protein quality control, intra- and extracellular propagation of peptide aggregates, disturbs in synaptic activity, and mitochondrial dysfunction (de Vrij et al., 2004; Selfridge et al., 2013; Marsh and Alifragis, 2018; Balupuri et al., 2020; Monteiro et al., 2020). Mitochondria are essential organelles for oxidative metabolism, and their loss of functionality is frequently associated with overall energetic failure (Liesa and Shirihai, 2013; Fang et al., 2019). Mitochondria’s integrity is regulated through three fundamental mechanisms like fusion, fission, and mitophagy. Their proper functioning acts as a protective barrier against oxidative stress resulting from mitochondrial damage (Ma et al., 2020). Here, we summarize the current evidence regarding the deregulation of these processes in different neurodegenerative disorders, including 1) Alzheimer’s disease (AD), 2) Parkinson’s disease (PD), 3) Huntington’s disease (HD), 4) Frontotemporal dementia (FTD), and 5) Amyotrophic lateral sclerosis (ALS).

Based on the above, the donation of mitochondria has recently moved into the center of attention, given its potential to replace damaged mitochondria with healthy ones (Hayakawa et al., 2016; Peruzzotti-Jametti et al., 2020). The magnitude of the evidence has led to the development of several therapeutic strategies, including minocycline (Investigators, 2006; Quintero et al., 2006; Antonenko et al., 2010; Howard et al., 2020), polyphenols, such as curcumin and resveratrol (Quintero et al., 2006; Sandoval-Acuna et al., 2014; Naoi et al., 2019; Pannu and Bhatnagar, 2019), vitamins (Etminan et al., 2005; Hiller et al., 2018; Marie et al., 2021), coenzyme Q10 (Shults et al., 2002), monoamine oxidase B inhibitors (Guay, 2006; Carradori et al., 2016), creatine (Matthews et al., 1999; Investigators, 2006; Investigators, 2008) and mitoquinone (MitoQ) (Doughan and Dikalov, 2007; Snow et al., 2010; Aimaiti et al., 2021) (For a more description, the reader is redirected to Table 1). Unfortunately, none of these approaches comprehensively addresses mitochondrial dysfunction, but rather its consequences. Therefore, we summarize the described mitochondrial transfer (MT) regulators, part of a complex enzymatic network that still represents a question mark. Furthermore, we analyzed the relevance of identifying “help-me” signals to modulate the activation of MT-associated enzymes endogenously.

**TABLE 1:** Current therapeutic strategies against mitochondrial dysfunction in neurodegenerative diseases.

Compound name	Advantages	Disadvantages	References
Minocycline	Antioxidant properties	Clinical trials do not confirm neuroprotective effects	Investigators (2006), Quintero et al. (2006), Antonenko et al. (2010), Howard et al. (2020)
	Reduces mitochondrial calcium overloading		
	Easily penetrate the blood-brain barrier		
Polyphenols (Curcumin and Resveratrol)	Antioxidants properties	Poor absorption and low bioavailability	Quintero et al. (2006), Sandoval-Acuna et al. (2014), Naoi et al. (2019), Pannu and Bhatnagar (2019)
	Induce mitochondrial biogenesis		
Vitamins (A, B3, D, E)	Antioxidant properties	Daily dosage and controversial results	Etminan et al. (2005), Hiller et al. (2018), Marie et al. (2021)
Coenzyme Q10 (CoQ10)	An enhancer of the electron transport chain activity	Therapeutic benefits are associated with high doses (2.4 g/day)	Shults et al. (2002)
Monoamine oxidase B inhibitors	Inhibitors of dopamine metabolism	Adverse effects, including confusion and hallucinations	Guay (2006), Carradori et al. (2016)
	Prevents free oxidative radical formation		
Creatine	Promotes mitochondrial ATP production	Clinical trials do not confirm neuroprotective effects	Matthews et al. (1999), Investigators (2006), Investigators (2008)
Mitoquinone (MitoQ)	Protection against oxidative damage by inhibiting lipid peroxidation	Clinical trials do not confirm neuroprotective effects	Doughan and Dikalov (2007), Snow et al. (2010), Aimaiti et al. (2021)

## 2 Abnormalities in mitochondrial dynamics and mitophagy in brain degeneration

Etiological aspects of here referred degenerative brain disorders, and their association with mitochondrial defects, are detailed in Table 2.

**TABLE 2:** Neurodegenerative-related mutations and their association with mitochondrial dysfunction.

Brain degenerative disorder	Protein (Gene)	Mitochondria-associated mutations	Mitochondrial deficiencies			Lesion sites	References
			MD	Mi	OD		
Huntington’s disease (AD)	Huntingtin [HTT]	HTT	✓	✓	✓	Corpus striatum (Basal ganglia)	Haun et al. (2013), Manczak and Reddy (2015), Guo et al. (2016), Yin et al. (2016)
Alzheimer’s disease (AD)	Amyloid precursor protein [APP]; Presenilin 1 [PSEN 1]; Presenilin 2 [PSEN 2]	APP	✓	✓	✓	Frontal lobes, Entorhinal cortex (Medial Temporal Lobe), Hippocampus	Fukui et al. (2007), Pinto et al. (2013)
Parkinson’s disease (PD)	Leucine Rich Repeat Kinase 2 [LRRK2]; Parkinsonism Associated Deglycase [PARK7]; PTEN-induced Kinase 1 [PINK1]; Parkin RBR E3 Ubiquitin protein ligase [PRKN]; Synuclein Alpha [SNCA]	LRRK2, PINK1	✓	X	✓	Substantia nigra (Basal ganglia)	Finn et al. (2013), Mortiboys et al. (2015), Yue et al. (2015)
Frontotemporal dementia (FTD)	C9 protein [C9ORF72]; Coiled-coil-helix-coiled-helix domain containing 10 [CHCHD10]; TDP-43 [TARDBP]; Microtubule-associated Protein Tau [MAPT]; Valosin-containing protein [VCP]; Charged multivesicular body protein 2B [CHMP2B]; Sequestosome 1 [SQSTM1]; Ubiquilin 1 [UBQLN1]	C9ORF72, 10 + 16 MAPT, CHCHD10	✓	X	✓	Frontal and temporal brain lobes	David et al. (2005), Fatouros et al. (2012), Bannwarth et al. (2014), Onesto et al., 2016; Esteras et al. (2017), Choi et al. (2019), Liu et al. (2020a), Liu et al. (2020b)
Amyotrophic lateral sclerosis (ALS)	Cu Zn superoxide dismutase 1 [SOD1]; C9 protein [C9ORF72]; TDP-43 [TARDBP]; Coiled-coil-helix-coiled-helix domain containing 10 [CHCHD10]; Fused in Sarcoma (FUS) RNA-binding protein [FUS]	SOD1, C9ORF72, TARDBP, FUS	✓	✓	✓	Motor cortex, spinal cord and brainstem	Perera et al. (2014), Tan et al. (2014), Moller et al. (2017), Choi et al. (2019), Smith et al. (2019), Dafinca et al. (2020)

**Abbreviations: MD, mitochondrial dynamics; Mi, Mitophagy; OD, oxidative damage. (✓) Pathological events are described in at least one of the mutations specified in the “Protein (Gene)” box.

### 2.1 Fission and fusion

Mitochondrial dynamics are multistep transitions that modify mitochondria’s spatial organization and functionality (Palikaras et al., 2015; Tilokani et al., 2018). Fission involves the exact and highly controlled fragmentation of impaired mitochondria, while fusion refers to the mechanism for constructing mitochondrial biomass from smaller mitochondrial units (Tilokani et al., 2018). The GTPase dynamic-related protein 1 (Drp-1) and mitochondrial fission protein 1 (Fis1) are essential enzymatic regulators in fission dynamics, controlling the constriction of mitochondrial membrane portions and inhibiting the fusion machinery (Tilokani et al., 2018).

Experiments performed in HD models such as R6/1 and HdhQ111/Q111 mouse neurons exhibit the same findings. Mice-derived R6/1 striatal neurons exhibit excessive Drp-1-dependent mitochondrial fragmentation and accumulation of oxygen free radicals (Cherubini et al., 2020). At the same time, HdhQ111/Q111 neurons express severe downregulation of fusion regulators like OPA1 and Mfn1/2 but also significant upregulation of fission markers, including Drp1 and Fis1 (Manczak and Reddy, 2015). Guo et al. studies complement these observations, whose findings reveal that striatal and spiny neurons carrying mutant huntingtin protein have significant mitochondrial fragmentation, increased motor deficit, and striatal disturbances in different HD mice models (Guo et al., 2016). Based on evidence suggesting that the pool of deficient huntingtin proteins is receptive to post-translational modifications (Haun et al., 2013), authors have proposed that molecular mechanisms such as S-nitrosylation aggravate mitochondrial fragmentation and provoke severe disorganization of dendritic spines by increasing its affinity with fission regulators (Haun et al., 2013). These observations have been convincingly demonstrated in BACHD rats-expressing human full-length huntingtin (Q97) and post-mortem brain samples (Haun et al., 2013).

In PD, nigral neurons have been broadly studied due to their dense mitochondrial biomass and oxidative activity, which make them especially susceptible to the consequences of mitochondrial dysfunction (Macdonald et al., 2018). Neurons exposed to toxic environmental substances like pesticides display considerable mitochondrial fragmentation and ATP depletion, correlating with the appearance of PD-like phenotypes *in vitro* (Chen et al., 2017). At the same time, mitochondria derived from LrrKG 2019S knock-in mice expressing a familial PD-related mutation are characterized by staying arrested in fission (Yue et al., 2015).

FTD-causing mutations, including C9orf72, 10 + 16 MAPT, and CHCHD10, have been connected with abnormalities in mitochondrial dynamics (Fatouros et al., 2012; Bannwarth et al., 2014; Onesto et al., 2016; Liu et al., 2020a; Liu et al., 2020b). Motor and mechanosensory neurons from *C. elegans* worms-expressing human Tau transgenes showed a significant redistribution of their mitochondrial network from the distal to the proximal region of axons (Fatouros et al., 2012), correlating with disturbances in the presynaptic region and locomotor deficiencies. Remarkably, Tau aggregation inhibitors reduce detergent-insoluble Tau aggregates, delay the accumulation of neuronal deficits, and promote a moderate improvement of locomotor abilities (Fatouros et al., 2012). Likewise, human fibroblasts carrying the C9orf72 mutation sustain abnormalities in mitochondrial fragmentation with a severe loss of ultrastructural features, the so-called cristae pattern (Onesto et al., 2016). At the same time, CHCHD10^S55L^ dopaminergic neurons have the same pathological findings accompanied by marked swelling (Anderson et al., 2019). Consistently, CHCHD10 mutation was reported since it provokes inhibition of mitochondrial fusion through dissociating OPA1-mitofilin complexes (Liu et al., 2020a).

An elevated number of FTD-related cases underlie the C9orf72 gene mutation, representing many genetic ALS cases (Ferrari et al., 2011). Additionally, ALS-related mutations might include those over Cu, Zn superoxide dismutase 1 (SOD1), CHCHD10, and TDP-43, which also cause various mitochondrial disturbances (Tan et al., 2014; Davis et al., 2018; Choi et al., 2019; Dafinca et al., 2020). In SOD1 mutant mice, the deregulation in IP3R/VDAC complexes has been associated with abnormalities in the interaction between the endoplasmic reticulum and mitochondria, leading to abnormal calcium dynamics, extensive mitochondrial fragmentation, and damage of ultrastructural features (Tan et al., 2014; Smith et al., 2019). Mice expressing SOD1A4V, SOD1G73R, and SOD1G93A mutations develop disorganization of mitochondrial networks in different types of neurons (Moller et al., 2017). By comparison, experiments conducted by Choi et al. support that C9orf72 mutants replicate most of these pathogenic features, such as glutamate-induced synaptic excitotoxicity and subsequent activation of apoptotic pathways (Choi et al., 2019; Dafinca et al., 2020).

### 2.2 Mitophagy

Mitophagy implies an autophagic pathway to recycle and reuse mitochondrial constituents (Palikaras et al., 2015). Although mitophagy undergoes a natural decline as aging progresses, numerous studies link the formation of protein aggregates and the dysregulation of intracellular pathways associated with mitophagy.

Experiments conducted by Tammineni et al. show that Aβ aggregates disturb the expression of specific molecular adaptors that regulate the identification, transport, and positioning of mitochondria through neuronal axonal projections (Tammineni et al., 2017), which is recognized as an event that precedes synaptic dysfunction. In AD, Aβ peptides and hyperphosphorylated forms of Tau aggregates provoke the downregulation of mitophagy-related proteins and deficiencies in lysosomal functionality, thus causing the accumulation of damaged mitochondria (Reddy and Oliver, 2019). Moreover, the long-term dysregulation of mitophagy exacerbates the appearance of mitochondrial DNA damage and provokes irreversible activation of apoptotic pathways (Fang et al., 2016).

In HD models, brain and skeletal muscle cells express elevated co-localization between autophagic/ubiquitination markers and mitochondrial constituents (Pinho et al., 2020). YAC128 and R6/2 mice carrying HD-like mutations acquire behavioral and motor abnormalities, which correlate with severe mitochondrial fragmentation and mitophagy in striatal and spiny neurons. Remarkably, blocking the interaction between mutant huntingtin and valosin-containing protein (VCP), a member of the AAA (+) ATPase family of chaperone-line proteins, reduces the mitochondrial translocation of the later, improves the mitochondrial organization in neurons and partially rescues disturbances in the behavior and motor abilities (Guo et al., 2016).

Lastly, ALS-associated TDP-43 mutations were recently documented by provoking evident deregulation of autophagy up-stream regulators like the AMP-activated protein kinase (AMPK) pathway (Perera et al., 2014; Smith et al., 2019); while mutations related to optineurin disrupt the recruitment of the autophagic machinery to the outer mitochondrial membrane and suspend its incorporation into autophagosome structures (Wong and Holzbaur, 2014).

## 3 Deregulation of mitochondrial complexes and oxidative stress in brain degeneration

Oxidative stress results from the uncontrolled generation of reactive oxygen species (ROS) like superoxide radicals (O_2_•-), hydrogen peroxide (H_2_O_2_), and hydroxyl radicals (•OH), thus exceeding the antioxidant defenses of cells and tissues (Pizzino et al., 2017). ROS are predominantly generated through mitochondrial respiration, obtaining O_2_•- molecules which are subsequently metabolized using intrinsic ROS scavenging enzymes, including superoxide dismutase (SOD), catalase, and glutathione peroxidase (Ray et al., 2012). Then, the coordinated metabolization of O_2_•- via SOD gives rise to the generation of H_2_O_2_, while radical hydroxyl molecules are generated through Fenton’s reaction, which entails the reaction between iron (Fe^2+^) and hydrogen peroxide (Fe^2+^ + H_2_O_2_ > Fe^3+^ + •OH + OH-) (Ray et al., 2012).

Numerous environmental and chemical stressors can exacerbate ROS production, including radiation, anti-blastic drugs, and exposure to heavy metals, among many others (Pizzino et al., 2017). Likewise, brain tissue differs from the rest, given its higher metabolic activity (Watts et al., 2018), providing a more significant potential to generate damage due to oxidative stress. Moreover, there is a natural age-related decline in mitochondrial functionality, which favors the accumulation of damaged mitochondria that generate high ROS amounts but lower ATP content (Hamilton et al., 2001). The following section focuses on how neurodegenerative conditions accelerate the functional decline of mitochondria, predisposing cells, and tissue to oxidative damage.

Experiments conducted in different research models, such as yeast (Bulteau et al., 2012), *Drosophila* (Greene et al., 2003), worms (Ren et al., 2019), zebrafish (Flinn et al., 2009), mice (Fukui et al., 2007; Pinto et al., 2013; Choi et al., 2019), and rats (Hoglinger et al., 2005; Escobar-Khondiker et al., 2007; Orozco-Ibarra et al., 2018), or even samples obtained from patients (Mahad et al., 2009; Mao and Reddy, 2010; Domercq et al., 2011; Mao et al., 2013; Sanders et al., 2014; Esteras et al., 2017; Igoillo-Esteve et al., 2020; Pistono et al., 2020), support that the expression and functioning of electron transport chain complexes are deregulated during brain degeneration (Hoglinger et al., 2005).

In AD, the metabolic profile seems to be a crucial determinant in generating amyloidogenic derivates since oxidative stress is tightly associated with the intensification in the Aβ peptide (AβPP) processing into Aβ (Gabuzda et al., 1994; Leuner et al., 2012). Experiments performed in cytoplasmic hybrids cells (or Cybrids) reveal that cytosolic constituents isolated from AD samples can replicate AD-like pathologic features once transplanted into healthy non-nucleated cells (Khan et al., 2000). These cells showed robust activation of proteolytic enzymes involved in regulating apoptotic pathways, increased DNA oxidative damage, poor ATP production, and strong activation of the amyloidogenic pathway, restored by antioxidant agents (Khan et al., 2000). The impact of the electron transport chain is evidenced through *in vivo* experiments crossing COXI mice carrying cytochrome c oxidase deficiencies and AD mice expressing human amyloid precursor protein (APP). Compared with AD mice, COXI/AD mice maintain reduced levels of the oxidative stress marker 8-hydroxy-2-deoxyguanosine (8-OHdG) and several Aβ deposits. At the same time, their mitochondrial DNA stability in the cortex and the hippocampus is significantly higher (Fukui et al., 2007; Pinto et al., 2013).

Most PD sporadic presentations are closely linked to defects in the expression and functionality of the mitochondrial complex I in the substantia nigra and the prefrontal cortex (Schapira et al., 1990; Parker et al., 2008). As expected, this deficiency type is primarily connected with energetic failure and progressive ROS accumulation (Greenamyre et al., 2001). Post-mortem analyses in the brain of PD-diagnosed patients support that nigral neurons carry extensive mitochondrial DNA damage (Sanders et al., 2014), and their cerebrospinal fluid contained elevated amounts of antioxidant co-enzyme Q10 and oxidized nucleosides (Isobe et al., 2010), revealing the presence of DNA lesions. Although most evidence attributes these alterations to the downregulation of mitochondrial complex I, experiments conducted by Schapira et al. in the early 1990s reported that PD patients do not display differences in the total protein and mitochondrial biomass neurons residing in the substantia nigra; however, their functionality at the level of complex I, is reduced (Schagger, 1995). Numerous investigations currently support these findings. For example, *Drosophila* (Greene et al., 2003) and zebrafish embryos (Flinn et al., 2009) carrying Parkin mutations show reduced functionality in complex I, while PINK1 mutants develop dysfunction in mitochondrial complexes I and III (Flinn et al., 2013). Although evidence consistently points to complex I dysfunction, the pathological contribution of complexes III subunit UQCR2, complex IV, and V subunit ATP5A have also been reported in a Lrrk^G2019S^ knock-in mouse (Mortiboys et al., 2015; Yue et al., 2015).

Striatal cells exposed to 3-nitro propionic acid, a chemical method for inducing HD-like striatal degeneration, proved to generate suppression of mitochondrial complex II and reduced ATP levels (Orozco-Ibarra et al., 2018). Notably, the isolation of mitochondria from neuronal synaptic terminals revealed variations in the protein expression of mitochondrial complexes, encompassing the upregulation of the complex II 70 kDa subunit (Hamilton et al., 2017), while neurons carrying huntingtin mutations (Hdh^Q111/Q111^) exhibit dysregulation of different mitochondrial-encoded electron transport chain subunits (Manczak and Reddy, 2015; Yin et al., 2016).

Comparably, neuron-like cells obtained via differentiation of FTD patient-derived iPSCs underline the consequences of the 10 + 16 MAPT mutation, which disrupts the mitochondrial membrane potential, reduces the activity of complex I, increase ROS generation and provoke cell death (Esteras et al., 2017). In agreement, six-month-old CamKII; (GR)80 mice express a GGGGCC repeat expansion within a non-coding region in the gene encoding for C9orf72. These mice evidence poor mitochondrial complexes I and V activity, severe DNA damage, and neuronal degeneration in the prefrontal, parietal, and occipital cortex. Also, these lesions correlate with synaptic abnormalities and FTD-like behavior (Choi et al., 2019). In contrast, mutations on TDP-43 provoke an aggressive phenotype characterized by abnormalities in the assembly and activity of complex I. This leads to severe neurotoxicity involving degeneration of cortical neurons and motor tracts, which is efficiently rescued by blocking the entrance of mutated TDP-43 protein into mitochondria (Wang et al., 2016). Similar observations were performed in P301L Tau transgenic mice. Proteomic and functional analyses showed that the generation of Tau aggregates destabilizes the activity of cytosolic upstream regulators for mitochondrial functions and antioxidant defenses (David et al., 2005). In agreement with both C9orf72 and TDP-43 mutants, these mice sustain the poor activity of mitochondrial complex I and low ATP content (David et al., 2005). In turn, the authors reported the dysregulation of different detoxifying enzymes, including glutathione reductase, glutathione peroxidase, and superoxide dismutase (David et al., 2005), pointing out that the enzymatic network that maintains mitochondrial homeostasis is altered at different levels.

Lastly, several ALS-associated mutations, including those shared with FTD, develop extensive oxidative damage. SOD1 mutated proteins are progressively stored at the mitochondrial intermembrane space, forming protein aggregates that lead to the deregulation of mitochondrial respiratory complexes and increased ROS generation (Smith et al., 2019). A comparable phenotype is observed in cells carrying mutations on the nuclear ribonucleoprotein P2, or FUS. These mutations are linked to uncontrolled ROS production and poor ATP biosynthesis. However, the mechanism breaks the interaction between the endoplasmic reticulum and mitochondria (Smith et al., 2019). On the other hand, neurons derived from C9orf72 transgenic mice sustain low activity in mitochondrial complexes I and V and severe DNA damage. As expected, these alterations converge in the rapid activation of apoptosis (Choi et al., 2019).

Altogether this data supports that the onset and progression of degenerative brain disorders entail enzymatic deregulation at different levels. The resolution of these defects has been focused through various methodologies, including i) nutritional supplements (Bobadilla et al., 2021; Dubey et al., 2021); ii) enzymatic replacement therapy based on soluble enzymes or even enzyme-loaded nanoparticles (Dickson et al., 2007; Del Grosso et al., 2019; Sato and Okuyama, 2020); and iii) mono-drug schemes (Andreux et al., 2013; Singh et al., 2021). Nevertheless, none of these methods can integrally correct mitochondrial dysfunction since they are directed against specific defects.

The following section discusses the potential of naturally occurring mitochondrial transfer as a therapeutic strategy expected to re-establish the functionality of mitochondria in neural cells undergoing energetic distress. By contrast with other therapeutic approaches, mitochondrial transfer offers the complete replacement of impaired mitochondria.

## 4 Spontaneous mitochondrial transfer: How does it happen, what is its relevance, and what role do enzymes play?

A growing body of evidence highlights the benefits of naturally occurring mitochondrial transfer, given its potential to restore mitochondrial dysfunction and energetic balance in neighboring cells.

### 4.1 Types of mitochondrial transfer (MT)

**FIGURE 1:**
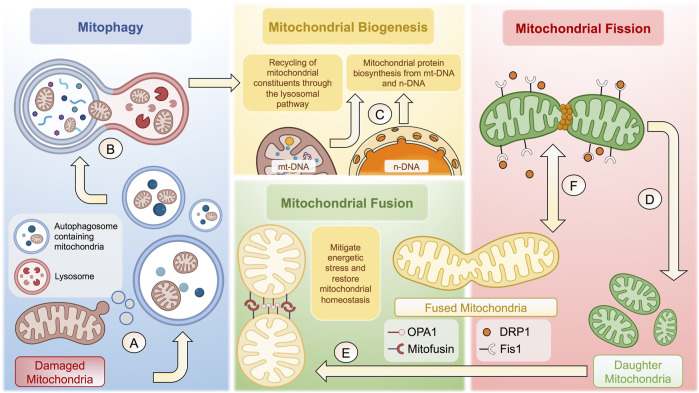
Cellular and molecular mechanisms behind mitochondrial homeostasis. **(A)** Damaged membrane microdomains of mitochondria are packaged in autophagosomes to follow the lysosomal pathway. **(B)** Autophagosomes containing mitochondria fuse with lysosomes to form autophagolysosomes to degrade mitochondria. **(C)** Once degraded, mitochondrial constituents are recycled to potentiate mitochondrial biogenesis together with proteins synthetized from n-DNA and/or mt-DNA. **(D)** Mitochondrial fission is tightly regulated through Fis1-dependent recruitment of DRP1, which generates a constriction ring to give rise to two daughter mitochondria. **(E)** Resulting mitochondria can undergo mitochondrial fusion to increase the mitochondrial biomass and thus mitigate energetic stress through OPA1 and Mitofusin proteins. **(F)** The balance between mitochondrial fusion and fission is highly dynamic, depending of various factors, including the bioenergetic status and cell-specific functions, among many others. The complexity of the regulation of mitochondrial homeostasis is not fully depicted. The figure was created in BioRender.com.

Defined as the naturally occurring transfer of mitochondrial units from a healthy donor to a specific cell acceptor with damaged mitochondria. Donor cells deliver mitochondria through different cellular mechanisms, including i) the formation of transient tunneling nanotubes (TNTs), which originate as filopodia-like cell membrane projections that extend, contact, and fuse with targeted cells (Gerdes et al., 2007; Caicedo et al., 2017); ii) the opening of intercellular channels located laterally in the membrane known as Gap junctions, by means cells exchange a variety of molecules as small-sized mitochondrial constituents or even energetic nucleotides (Li et al., 2019); and iii) the release of extracellular vesicles (EVs) that carry mitochondrial constituents of different sizes (Hayakawa et al., 2016; Peruzzotti-Jametti and Pluchino, 2018). For example, microvesicles ranging between 500 and 900 nm can transport entire mitochondrial units (Hayakawa et al., 2016). In comparison, those oscillating between 50 and 100 nm can carry small mitochondrial microdomains containing respiratory complexes and other integral membrane elements (Peruzzotti-Jametti et al., 2020). Figure 1.

### 4.2 MT-associated enzymatic regulators

**FIGURE 2:**
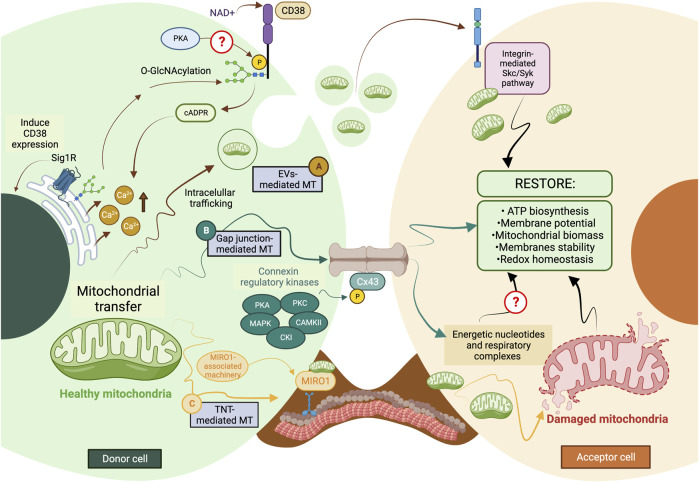
The current model of cellular and molecular mechanisms behind mitochondrial transfer. **(A)** EVs-mediated MT is regulated through the CD38/cADPR signaling, inducing calcium release from the endoplasmic reticulum. The CD38 activity can be post-translationally modulated through O-GlcNAcylation and phosphorylation, and its expression is induced in a Sig1R-dependent manner. **(B)** Connexins’ protein expression, assembly, and stabilization are precisely controlled through different kinases, such as PKA, PKC, MAPK, CK1, and CAMKII. Gap junctions-mediated MT is predicted to facilitate the donation of low-weight molecules comprising energetic nucleotides and mitochondrial respiratory complexes. **(C)** The formation of TNTs relies on the molecular interaction between MIRO1 and accessory motor enzymes like dynein, kinesins, and myosins. TNT-mediated MT entails donating complete mitochondrial units, whose subcellular fate is still the subject of discussion. The complexity of the exocytic pathway is not fully depicted. Question marks denote mechanisms not currently described. MT, mitochondrial transfer; PKA, protein kinase A; PKC, protein kinase C; MAPK, mitogen-activated protein kinase (MAPK); CK1, casein kinase 1; and CAMKII, Ca^2+^/calmodulin-dependent kinase II. The figure was created in BioRender.com.

Most mechanisms behind regulating mitochondrial transfer and their crosstalk are still poorly understood (Figure 2). In addition, current enzymatic functions are detailed in Table 3.

**TABLE 3:** Current roles of mitochondrial transfer-related enzymes.

Enzyme	MT-associated functions	Enzymatic network	References
Rho GTPase 1 (Miro1)	It regulates TNTs formation, functioning as a molecular adaptor that connects the mitochondrial membrane and microtubule motor proteins to facilitate the displacement of mitochondria	Myosins, kinesins, dynein	Utton et al. (2005), Boukelmoune et al. (2018), Oeding et al. (2018), Tseng et al. (2021)
Cluster of Differentiation 38 (CD38)	It degrades NAD + molecules for activating calcium-dependent pathways that act as initiation steps for the biogenesis of EVs containing mitochondria	Sig-1R, ERK1/2, PKA	Lee, 2011; Wei et al. (2014), Hayakawa et al. (2016), Wang et al. (2020), Park et al. (2021)
Src and Syk kinases	Participate in the incorporation of EVs containing mitochondria	n.d.	Hayakawa et al. (2016)
Connexin-related enzymes	They regulate the expression, subcellular distribution, and assembly of connexins into connexons. Once assembled, connexins work as railways for trafficking mitochondrial constituents	CaMKII, MAPK, PKA, PKC, CK1	Axelsen et al. (2013), Islam et al. (2012), Ren et al. (2022)

n.d., non-described.

During TNT’s formation, mitochondrial Rho GTPase 1, also known as MIRO1, mediates the polymerization of actin filaments and the subsequent establishment of intercellular structures connecting the donor and acceptor cells (Lopez-Domenech et al., 2018). This GTPase functions as a molecular adaptor that binds the mitochondrial outer membrane and microtubule motor proteins, thus facilitating the displacement of mitochondria along microtubules (Lopez-Domenech et al., 2018). MIRO1 gain-and-loss of function experiments resulted in changes in MT’s efficiency and repair capacity (Boukelmoune et al., 2018; Tseng et al., 2021).

CD38 (Cluster of Differentiation 38) is a multifunctional ectoenzyme that controls extracellular nucleotide homeostasis and intracellular calcium oscillations (Hogan et al., 2019). CD38 degrades NAD + molecules as a cyclase to produce cyclic ADP-ribose (cADPR) molecules, a potent second messenger for calcium mobilization (Hogan et al., 2019). The release of EVs containing mitochondrial units and constituents depends on the CD38/cADPR axis (Hayakawa et al., 2016). Furthermore, cellular states like oxygen-glucose deprivation and reoxygenation induce changes in the post-translational modification of mitochondrial proteins via O-GlcNAcylation (O-GlcNAc), increasing the release of EVs containing mitochondria in a CD38-dependent manner (Park et al., 2021). Consistently, the genetic inhibition of CD38 reduces mitochondrial transfer *in vitro* and *in vivo* (Hayakawa et al., 2016); defects in the endoplasmic reticulum–Golgi traffic resulted in the release of mitochondria with reduced membrane potential and mt-DNA (Park et al., 2021). Since O-GlcNAc has been previously implicated in protein quality control (Collins and Chatham, 2020), it is tempting to speculate whether mitochondria undergo a pre-selection before being charged and released. Little is known about the internalization of EVs carrying mitochondria, but it entails the activation of the integrin-mediated Src/Syk signaling pathway (Hayakawa et al., 2016) (Figure 2).

### 4.3 Targeting possible MT-associated enzymes

#### 4.3.1 MIRO1-associated enzymes

MIRO1-mediated MT relies on the synchronized interaction of many enzymes involving myosins, dynein, and the kinesin superfamily proteins (KIFs). A proposal of their interaction during MT is detailed in Figure 2, while their molecular mechanisms are summarized in Table 3.

Myosins are molecular motors that produce mechanical energy through ATP consumption, comprising a superfamily classified into 18 classes (Foth et al., 2006). These proteins contain C-terminal light chain-associated domain, which is regulated through phosphorylation to modulate myosin’s activity (Sata et al., 1997). Furthermore, myosins contain motor domains that catalyze the hydrolysis of ATP. The direct interaction between myosin and MIRO1 facilitates actin-based mitochondrial transport (Oeding et al., 2018).

KIFs are motor proteins whose classification relies on the location of their motor domains, comprising N- (N-terminal), C- (C-terminal), and M- (Middle portion) kinesins (Rath and Kozielski, 2012). These enzymes participate in various cellular processes such as intracellular traffic dynamics, cell division, and the anterograde transport of mitochondria through axons (Hirokawa et al., 2009). Mechanistically, KIFs intervene in the binding of motor proteins and mitochondria, and their loss of function, specifically on the kinesin-related protein 5 (KIF5), disturbs the traffic of mitochondria and correlates with abnormalities in their subcellular location (Utton et al., 2005).

Dynein is a minus-end-direct motor enzyme whose ATP consumption displaces mitochondria across the cell body, distributed in axonal and cytoplasmic isoforms (Gibbons and Rowe, 1965). Dynein functionality is subject to the cofactor dynactin, a 23-subunit complex that supports the retrograde transport of cellular constituents, including mitochondria (Urnavicius et al., 2015). The genetic inhibition of dynein ameliorates its binding to mitochondria and gives rise to cytoplasmic inclusions that interfere with mitochondria’s transport in neurons (Chen et al., 2014).

#### 4.3.2 CD38-associated enzymes

The topology of CD38 is still a matter of discussion. It is predicted to be a type-II transmembrane protein with a catalytic C-terminal domain pointing to the extracellular media (Wei et al., 2014). This presents a dilemma because the NAD substrate is stored intracellularly. Cells co-express different types of CD38, which differ in the subcellular distribution and structurally (Zhao et al., 2012). In this hypothesis, some catalytic domains should be oriented outside the cell, while others should be disposed to the cytoplasm. In this scheme, the catalytic C-terminal domain takes relevance since it contains multiple serine (Ser) residues, among which a phosphorylation site for protein kinase A (PKA) is predicted to exist (Lee, 2011; Wei et al., 2014).

Sigma-1 receptor (Sig-1R), one of two sigma receptor subtypes, is a 223-amino-acid-long trans-membrane chaperone at the endoplasmic reticulum (Zhemkov et al., 2021). Mechanistically, the activation of Sig-1R upregulates the expression of CD38 through extracellular regulated protein kinases 1/2 (ERK1/2), facilitating the CD38-driven MT. Knocking down CD38 abolishes Sig-1R-induced MT (Wang et al., 2020).

#### 4.3.3 Connexons-related enzymes

The stabilization and maintenance of Gap junctions entail the synchronic displacement of connexins along the plasma membrane, laterally assembled into connexin hemichannels, known as connexons (each one formed by six connexins) (Zimmermann, 1984). Gap junctions’ formation is coordinated through several phosphatases and kinases (Solan and Lampe, 2016). The specific phosphorylation of serine (Ser) and tyrosine (Tyr) residues in connexins instruct their correct displacement and assembly into connexons (Solan and Lampe, 2016). Some sites of phosphorylation and their specific kinase include 1) Ser^244^ and Ser^314^ for Ca^2+^/calmoduline-dependent kinase II (CaMKII); 2) Tyr^247^ and Tyr^265^ for Src kinase; 3) Ser^255^, Ser^279^, and Ser^282^ for Mitogen-activated protein kinase (MAPK); 4) Ser^262^ for protein kinase C (PKC); 5) Ser^325^, Ser^328^, and Ser^330^ for casein kinase 1 (CK1); and 6) Ser^364^, Ser^365^, Ser^368^, Ser^369^, Ser^372^, and Ser^373^ for PKA and PKC [Reviewed in (Axelsen et al., 2013)]. Their possible interactions are proposed in Figure 2.

### 4.4 MT in brain degeneration

Different types of cells employ spontaneous mitochondrial transfer in the central and peripheral nervous systems, including various subtypes of neurons (Li et al., 2019; English et al., 2020; van der Vlist et al., 2022), astrocytes (Hayakawa et al., 2016; English et al., 2020), neural and endothelial progenitor cells (Hayakawa et al., 2018), and nervous tissue-residing immune cells (Luz-Crawford et al., 2019; Court et al., 2020). Although the incorporation mechanisms remain poorly detailed, host cells recover their survival and mitochondrial functionality by increasing oxygen consumption and ATP generation (Hayakawa et al., 2016). Cells carrying mitochondrial impairment incorporate approximately 40% more biomass than healthy acceptor cells (English et al., 2020); current evidence suggests that donor cells can transfer between 5% and 13% of their whole mitochondrial biomass (Hayakawa et al., 2018; Gao et al., 2019), allowing them to remain viable. Both proteomic and gene ontology determinations support that adult neural progenitors can transfer mitochondrial complexes encoded in mitochondrial and nuclear genomes (Peruzzotti-Jametti et al., 2020). The purification and subsequent intravenous administration of mitochondria increase the expression of antioxidant enzymes, reduce lipid peroxidation, limit the generation of oxygen and nitrogen-derived reactive species in oxygen-deprivation conditions, and increase neuronal survival (Zhang et al., 2019).

In the host tissue, MT modulates pro-angiogenic properties, endothelial permeability, neuronal survival in front of oxygen and glucose deficiency, and inflammatory pain resolution, as well as the restriction of reactive astrogliosis and the induction of adult neurogenesis (Bond et al., 2015; Hayakawa et al., 2018; Zhang et al., 2019; van der Vlist et al., 2022). Even neuroglial transmitophagy represents an MT-based mechanism through which astrocytes internalize and degrade axonal mitochondria (Lampinen et al., 2022), which can induce neuroprotection in pathological contexts (Hayakawa et al., 2016; English et al., 2020). Analogously, this mechanism is also employed by cone photoreceptors. These retinal neurons can transfer injured mitochondria from the cone to Müller glia to be degraded (Hutto et al., 2023).

The therapeutic potential of MT in degenerative brain disorders is still a growing field of research. Mice expressing 1-Methyl-4-phenil-1,2,3,6-tetrahydropyridine (MTPT)-induced PD-like phenotype respond positively to mitochondria’s administration, improving their locomotor and behavioral abilities (Zhang et al., 2019). Zhang et al. tested the impact of muscle-derived mitochondria once intraventricularly injected into rats’ brains. They showed that mitochondria confer strong resistance against oxidative stress and significantly increase mitochondrial biomass after injury (Zhang et al., 2019). In addition, AD mice intravenously injected with mitochondria improve their cognitive performance, which correlates with a significant increase in neurons’ survival and further restriction of reactive astrogliosis (Nitzan et al., 2019). These effects are subjected to Gap-junctions-mediated MT. Activating the GJA1-20K/connexin 43 (Cx43) axis rescues the dendrite length and promotes mitochondrial protein expression in neurons after brain injury through the spontaneous transfer of mitochondria from astrocytes (Ren et al., 2022). Consistently, the loss of MT from astrocytes to neurons has also been documented in toxic-induced cognitive impairment and leukodystrophies (Gao et al., 2019; English et al., 2020).

### 4.5 What about the pathological microenvironment?

Differences in preserving or declining the integrity of certain neuroanatomic regions through brain degeneration remain a long-standing question (Morrison et al., 1998; Mrdjen et al., 2019). Cellular and molecular differences between CNS-residing cells seem to explain the vulnerability or resistance against stressor factor, such as secretory and biosynthetic demands, oxidative stress, misfolding and aggregation of proteins, calcium fluxes, glucose and oxygen restriction, and nervous and/or peripheral inflammatory responses (Mrdjen et al., 2019). Moreover, aging gradually impairs the capacity of cells to deal with these stressors (Mrdjen et al., 2019).

The neuroanatomical perspective takes strength due to the specific pattern of cell decline for each disorder, involving corticocortical neurons in AD, cingulate and insular neurons in FTD, upper and lower motor neurons in ALS, striatal neurons in HD, and nigrostriatal neurons in PD (Morrison et al., 1998; Nana et al., 2019). In this regard, the differential expression of neurofilament proteins and neuron-specific receptors has been proposed as major vulnerability mechanisms. For example, glutamatergic neurons expressing N-methyl-D-aspartate (NMDA) receptors are highly vulnerable to voltage-gated Ca^2+^ channel-dependent excitotoxicity (Morrison et al., 1998).

Although the chronic course of degenerative brain disorders compromises other brain regions, there are areas with high resistance that remain intact even in advanced stages (Mrdjen et al., 2019). Thus, stimulating endogenous MT could represent a complementary method to induce the regeneration of diseased brain regions from healthy ones. Even it may cover some technical aspects associated with the exogenous administration of mitochondria, a research field with promising advances (McCully et al., 2009; Masuzawa et al., 2013; Cowan et al., 2016; Emani et al., 2017; Shi et al., 2017).

### 4.6 Experimental and biological considerations

Biodistribution studies show that nervous cells store significant amount of mitochondria after being intravenously administered, increasing ATP biosynthesis, restoring the energetic failure and thus promoting survival in recipient cells (Shi et al., 2017). Nevertheless, some experimental considerations should be covered when studying the therapeutic potential of isolated mitochondria.

Mitochondria’s onion-like organization plays a fundamental role in maintaining mitochondria’s functionality, favoring the establishment of cristae-like structures that increase the reaction surface. Mitochondrial swelling is a morphological feature resulting from the opening of the permeability transition pore, the entry of water, and then the disorganization of cristae structures (Javadov et al., 2018), representing a hallmark of mitochondrial dysfunction. Mitochondria are isolated using centrifugation steps aimed at separating them from the rest of cellular components (Caicedo et al., 2015), and to be subsequently added to cell cultures, and injected directly into the tissue or circulation (McCully et al., 2009; Caicedo et al., 2015; Cowan et al., 2016; Kaza et al., 2017; Shi et al., 2017). Since these environments do not preserve the osmolarity of the intracellular microenvironment given that calcium oscillates in micromolar concentrations (Valentine et al., 2018), it is interesting to speculate about how intact remain mitochondria once transferred outside the cell. Current evidence show that isolated mitochondria remain functional and conserve their ultrastructural organization (Caicedo et al., 2015; Peruzzotti-Jametti et al., 2020). However, our knowledge on the route they follow after being incorporated into cells is largely unknown. Perhaps, these mitochondria become dysfunctional on the way to be incorporated but still contribute to the energetic failure in recipient cells by acting as a substrate for the recycling of components needed to initiate mitochondrial biogenesis.

Following the line of structural integrity, immune system activation arises as a possible pitfall. Despite MT induces anti-inflammatory responses in different tissues (Luz-Crawford et al., 2019; van der Vlist et al., 2022), the loss of mitochondria’s integrity could derive in the release of mitochondrial DNA (mt-DNA). Several pathological conditions including oxidative damage, genotoxic stress, activation of pro-inflammatory factors and mitochondrial dysfunctions favor the release of mt-DNA (Kim et al., 2023). Once released, mt-DNA is recognized as a strong agonist of innate immunity, activating pro-inflammatory pathways such as endosomal localized TLR9, cytosolic cGAS-STING, and cytosolic inflammasome AIM2/NLRP3 (Riley and Tait, 2020). Therefore, experimental approaches that poorly preserve mitochondrial integrity through the isolation step could generate results conditioned by the activation of the immune system, but not associated with the incorporation of functional mitochondria.

Spontaneous mitochondrial transfer occurs through the formation of TNTs and connexons and the release of EVs. The deepening into MT-associated molecular and cellular mechanisms, and the development of strategies aimed at modulating them could represent an alternative to the osmolarity and immune considerations since bypass the exposure of mitochondria to the extracellular medium.

## 5 Conclusion and perspectives: looking for “help-me” signals

Most pharmacologic and genetic approaches aimed to modulate the MT-related enzymatic machinery are oriented to its inhibition since states of overactivation correlate with the appearance of pathological features (Braicu et al., 2019; Nassal et al., 2020). This represents a limitation given the need to enhance its function to induce mitochondrial transfer, calling to identify new mechanisms to activate endogenous MT.

Neural cells employ various “help-me” signals (i.e., cell-derived molecules released when cells undergo different types of damage, including mitochondrial damage) able to modulate neuroprotective mechanisms, such as regulating neurogenic and angiogenic pathways. These mechanisms include the release of mitochondrial debris, growth factors, chemokines, and cytokines, which have been extensively reviewed in the literature (Xing and Lo, 2017). Once released, these extracellular signals recruit neighboring cells, potentiate neuroprotection and promote endogenous brain regeneration (Xing and Lo, 2017). There is no evidence regarding variations in the secretome of cells with energy failure, not at least in neural cells. However, mitochondrial damage-associated molecular patterns (DAMPs) are conserved in mammals (Nakahira et al., 2015; Grazioli and Pugin, 2018). These patterns involve the release of mt-DNA and mitochondrial proteins from damaged cells, mediating the activation of diverse cellular mechanisms in their environment or even distant niches as they enter circulation (Nakahira et al., 2015; Grazioli and Pugin, 2018). On the other hand, experiments performed in epithelial, adipose, and fibroblast cells suffering dysfunction in oxidative phosphorylation revealed significant variations in their secretome (Llobet et al., 2017; Garrido-Perez et al., 2020). Molecules associated with focal adhesion, complement, and coagulation cascades, extracellular matrix receptors, glucose transporters, intracellular trafficking proteins, and hormones related to regulating appetite and satiety in the hypothalamus presented significant variations (Llobet et al., 2017; Garrido-Perez et al., 2020; Sturm et al., 2023). Besides, the secretion of cytokines and metabokines also suffers modifications in cells with age-related mitochondrial dysfunction. The progressive decline in mitochondria’s functionality is closely associated with a hypermetabolic state presenting significant mt-DNA instability and elevated secretion of growth differentiation factors (Sturm et al., 2023). Lastly, ROS overproduction represents a critical stimulus. It has been described that rotenone-induced oxidative damage potentiates MT efficiency, which is inhibited through administrating ROS scavenger molecules (Jiang et al., 2016; Burt et al., 2019). In this regard, NADPH oxidase-2-derived superoxide radicals are critical. Marlein et al. reported that blocking the generation of superoxide species abolishes MT in myeloid leukemia blasts (Marlein et al., 2017).

Based on the above data, an exciting projection regarding MT-related enzymes could rely on identifying “help-me” signals from cells undergoing bioenergetic stress. Indeed, there is little evidence regarding the regenerative potential of MT in degenerative brain disorders. However, both *in vitro* and *in vivo* determinations, associated or not with the nervous system, suggest that this is a promising tool to promote the endogenous revitalization of damaged tissue. Going deeper into identifying “help-me” signals could put us one step ahead of genetic or pharmacological approaches to modulate MT-related enzymes.
