# Corrigendum: Exploring the therapeutic potential of the mitochondrial transfer-associated enzymatic machinery in brain degeneration

**DOI:** 10.3389/fphys.2023.1278208

**Published:** 2023-08-23

**Authors:** Noymar Luque-Campos, Ricardo Riquelme, Luis Molina, Gisela Canedo-Marroquín, Ana María Vega-Letter, Patricia Luz Crawford, Felipe A. Bustamante-Barrientos

**Affiliations:** ^1^ Laboratorio de Inmunología Celular y Molecular, Facultad de Medicina, Universidad de los Andes, Santiago, Chile; ^2^ Centro de Investigación e Innovación Biomédica, Universidad de los Andes, Santiago, Chile; ^3^ IMPACT-Center of Interventional Medicine for Precision and Advanced Cellular Therapy, Santiago, Chile; ^4^ Escuela de Nutrición y Dietética, Facultad de Medicina, Universidad de los Andes, Santiago, Chile; ^5^ Facultad de Medicina y Ciencia, Universidad San Sebastián, Puerto Montt, Chile; ^6^ Faculty of Dentistry, Universidad de los Andes, Santiago, Chile; ^7^ Escuela de Ingeniería Bioquímica, Pontificia Universidad Católica de Valparaiso, Valparaiso, Chile

**Keywords:** degenerative brain disorders, mitochondrial dysfunction, fission and fusion, mitophagy, oxidative damage, mitochondrial transfer, cellular therapy, enzymes

In the published article, there was an error in **Affiliation** number 7. Instead of “Escuela de Ingeniería Bioquímica, Pontificie Universidad Católica de Valparaiso, Valparaiso, Chile,” it should be “Escuela de Ingeniería Bioquímica, Pontificia Universidad Católica de Valparaiso, Valparaiso, Chile.” “Pontificie” should be “Pontificia.”

The authors apologize for this error and state that this does not change the scientific conclusions of the article in any way. The original article has been updated.

